# Supplementary material for: Novel Driver Strength Index highlights important cancer genes in TCGA PanCanAtlas patients
Source: PeerJ. 2022 Aug 11;10:e13860. doi: 10.7717/peerj.13860 (PMC9375969; doi:10.7717/peerj.13860)
Supplement: Supplemental Information 2 [file peerj-10-13860-s002.zip › COHORTS/genes plots/cohorts/2021_8_16_14_9_distribution_cohorts_females_ESCA.pdf]

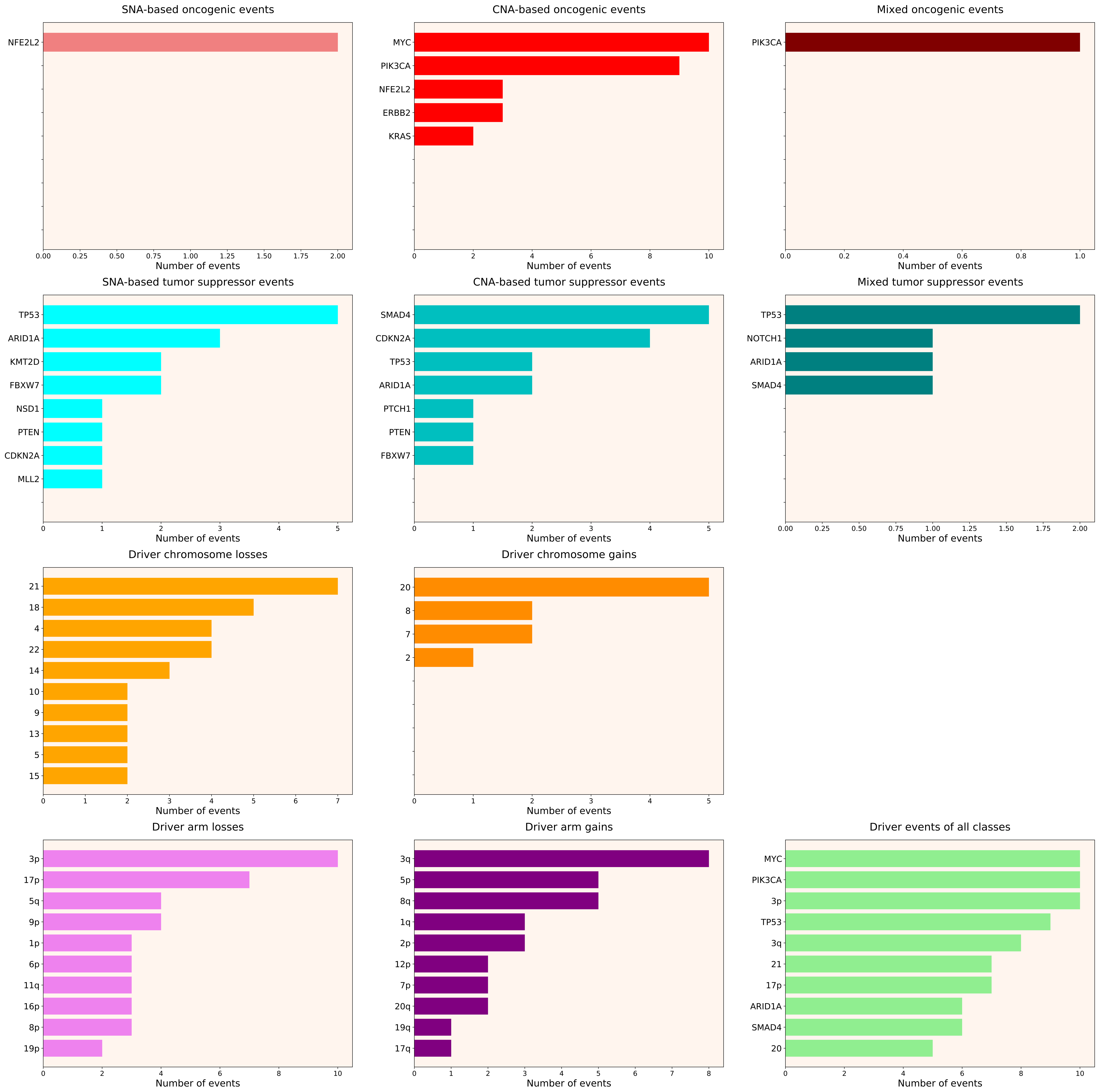
SNA-based oncogenic events
NFE2L2
0.00 0.25 0.50 0.75 1.00 1.25 1.50 1.75 2.00
Number of events
CNA-based oncogenic events
MYC
PIK3CA
NFE2L2
ERBB2
KRAS
0 2 4 6 8 10
Number of events
Mixed oncogenic events
PIK3CA
0.0 0.2 0.4 0.6 0.8 1.0
Number of events
SNA-based tumor suppressor events
TP53
ARID1A
KMT2D
FBXW7
NSD1
PTEN
CDKN2A
MLL2
0 1 2 3 4 5
Number of events
CNA-based tumor suppressor events
SMAD4
CDKN2A
TP53
ARID1A
PTCH1
PTEN
FBXW7
0 1 2 3 4 5
Number of events
Mixed tumor suppressor events
TP53
NOTCH1
ARID1A
SMAD4
0.00 0.25 0.50 0.75 1.00 1.25 1.50 1.75 2.00
Number of events
Driver chromosome losses
21
18
4
22
14
10
9
13
5
15
0 1 2 3 4 5 6 7
Number of events
Driver chromosome gains
20
8
7
2
0 1 2 3 4 5
Number of events
Driver arm losses
3p
17p
5q
9p
1p
6p
11q
16p
8p
19p
0 2 4 6 8 10
Number of events
Driver arm gains
3q
5p
8q
1q
2p
12p
7p
20q
19q
17q
0 1 2 3 4 5 6 7 8
Number of events
Driver events of all classes
MYC
PIK3CA
3p
TP53
3q
21
17p
ARID1A
SMAD4
20
0 2 4 6 8 10
Number of events
